# Intrauterine intestinal obstruction in a preterm infant with severe mevalonate kinase deficiency – a case report

**DOI:** 10.1186/s40748-025-00207-w

**Published:** 2025-03-05

**Authors:** Henrike Hoermann, Julia Franzel, Juliane Tautz, Prasad T. Oommen, Elke Lainka, Ertan Mayatepek, Thomas Hoehn

**Affiliations:** 1 Department of General Paediatrics, Neonatology and Paediatric Cardiology, Medical Faculty, University Hospital Duesseldorf, Heinrich Heine University, Moorenstraße 5, Duesseldorf, 40225 Germany; 2 Department of Paediatric Oncology, Haematology and Clinical Immunology, Medical Faculty, Division of Paediatric Rheumatology, University Hospital Duesseldorf, Heinrich Heine University, Moorenstraße 5, Duesseldorf, 40225 Germany; 3 Children’s Hospital, Department of Paediatric Gastroenterology, Hepatology, and Transplant Medicine, University Duisburg-Essen, Hufelandstraße 55, Essen, 45147 Germany

**Keywords:** Mevalonate kinase deficiency, Mevalonic aciduria, Intestinal obstruction, Prematurity

## Abstract

**Background:**

Mevalonate kinase deficiency is an inherited autoinflammatory disorder that can present with a wide clinical spectrum, ranging from mild forms with recurrent episodes of fever, lymphadenopathy, splenomegaly and skin rash to the much rarer severe form, which is characterized by additional occurrences of psychomotor impairment, cholestatic jaundice, ophthalmological symptoms, and failure to thrive. The few cases described with perinatal onset often showed a very severe clinical course.

**Case presentation:**

Here, we report the case of a preterm infant born at 30 + 2 weeks of gestation with a prenatal genetic diagnosis of mevalonate kinase deficiency presenting with intrauterine bowel dilatation, mild hydrops fetalis, and microcephaly. Laparotomy on the second day of life revealed intestinal obstruction necessitating partial ileum resection and ileostomy. The neonate had recurrent inflammatory reactions with elevated C-reactive protein levels, severe cholestasis, a progressive liver dysfunction, and an increasingly distended abdomen with subsequent respiratory insufficiency. Urinary mevalonic acid was highly elevated. The patient received anti-inflammatory therapy with prednisone and anakinra. Unfortunately, the patient died at the age of 77 days due to cardiorespiratory failure.

**Conclusions:**

This case shows that intestinal obstruction with dilated fetal bowel loops can be an initially leading clinical symptom of severe mevalonate kinase deficiency. Diagnostics should be considered at an early stage, especially in the presence of other anomalies such as hydrops fetalis, growth restriction, or microcephaly. Data on the neonatal course of severe mevalonate kinase deficiency are still scarce and further studies are needed, particularly on treatment in neonates and young infants.

## Background

Mevalonate kinase deficiency (MKD) is an autosomal recessive inherited disorder caused by mutations in the mevalonate kinase (MVK) gene on chromosome 12q24 [1, 2]. MVK is part of the isoprenoid biosynthesis pathway [3]. Its deficiency leads to a shortage of isoprenoid end-products and an accumulation of mevalonic acid [2, 3]. Depending on the residual function of the enzyme the clinical spectrum varies widely from mild forms (formerly known as hyperimmunoglobulinemia D syndrome (HIDS)) to severe forms such as mevalonic aciduria (MA) [4]. Following the “Consensus proposal for taxonomy and definition of the autoinflammatory diseases (AIDs): a Delphi study” by Ben-Chetrit et al. we used the term severe MKD for MA [5]. Typical symptoms of mild MKD are recurrent episodes of fever which can be accompanied by cervical lymphadenopathy, skin rash, joint, muscle and abdominal pain, splenomegaly, headache, vomiting and diarrhea [6]. Severe MKD is a multisystemic disease including mild to severe psychomotor impairment, cerebellar atrophy, ataxia, progressive proximal myopathy, facial dysmorphia (including microcephaly, triangular-shaped face, down-slanting eyelids, dysplastic ears), cataract, uveitis, retinitis, cholestatic jaundice and failure to thrive [6–9].

There are only a few cases where prenatal abnormalities such as hydrops fetalis or growth restriction are described in the context of severe MKD [7, 10–12]. Here we describe the severe clinical course of a preterm infant prenatally diagnosed with MKD presenting with severe intrauterine intestinal obstruction.

## Case

The female preterm infant was born at 30 + 2 weeks of gestation via cesarean section, which was indicated due to maternal fever and fetal tachycardia. Antenatal betamethasone was administered seven and eight days prior to delivery. Amniocentesis with whole-exome-sequencing was performed at 20 + 1 weeks of gestation due to fetal bowel dilatation, microcephaly (head circumference < 5^th^ percentile), and mild hydrops fetalis with ascites and pericardial effusion. The patient was compound heterozygous for the variants c.500C > T (p.Pro167Leu) (classified as likely pathogenic) and c.420_421dup (p.Ala141GlyfsTer19) (classified as pathogenic) in the MVK gene. The mutation c.420_421dup (p.Ala141GlyfsTer19) was detected in heterozygous form in the mother and the mutation c.500C > T (p.Pro167Leu) was detected in heterozygous form in the father. The parents are non-consanguineous. Genetic counselling was conducted prenatally. The mother is a second gravida, second para. The 2.5-year-old brother is healthy, a urine analysis of the brother revealed normal mevalonic acid concentrations. There are no other known cases with MKD in the family.

**Fig. 1:**
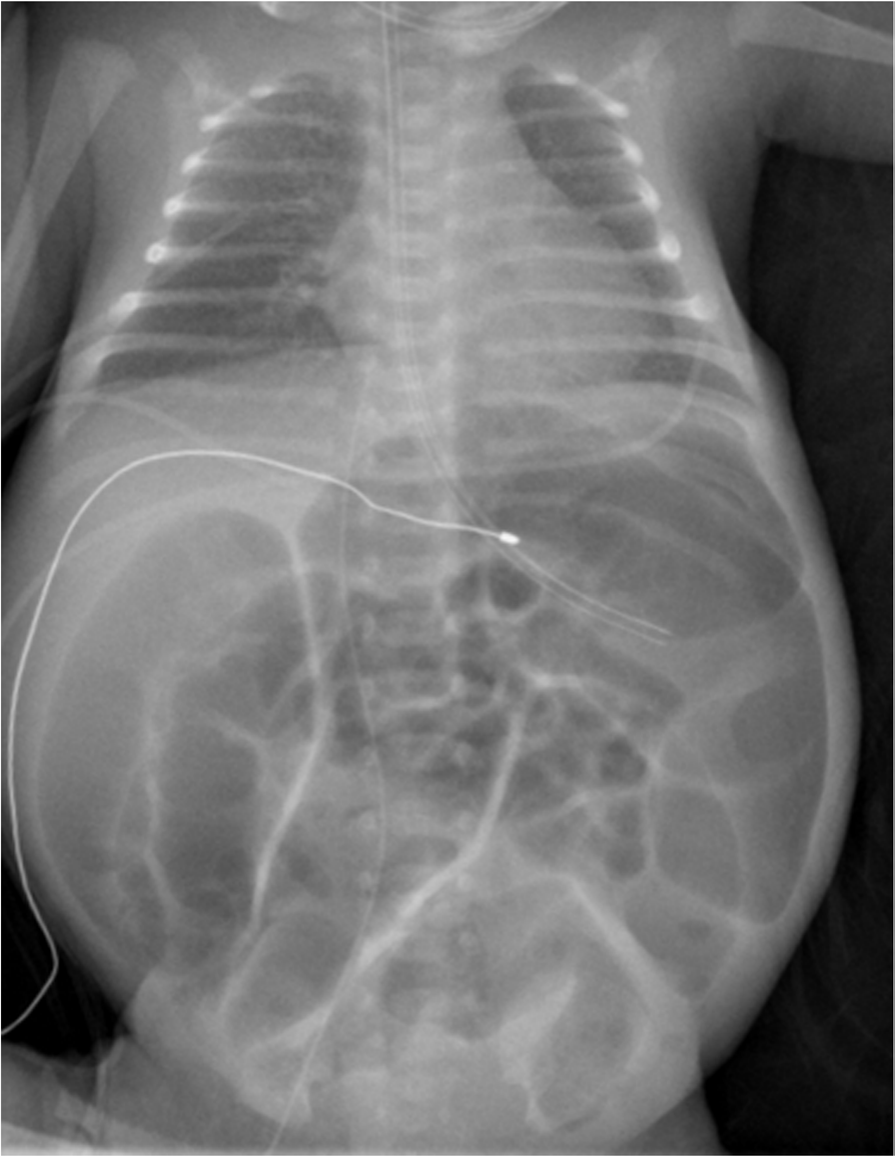
Chest and abdominal X-ray. The X-ray was taken on day of life two before surgery. It shows markedly distended intestinal loops but no sign for perforation or intraluminal gas

The preterm infant was born with a birth weight of 1240 g (29^th^ percentile), length 34 cm (4^th^ percentile) and head circumference 26.5 cm (19^th^ percentile). Voigt percentiles were calculated for birth measures [13]. Arterial umbilical cord blood pH was 7.3, Apgar score was 6/8/9. The preterm infant received continuous positive airway pressure and less-invasive surfactant administration was performed during the first hour of life. A C-reactive protein (CRP) of 8 mg/dL (norm: < 0.5 mg/dL) in venous blood from the child and dilated bowel loops were found immediately after birth. White blood cell count was 24,400/µl (norm: 6000–13,300/µl) and Interleukin 6 was 136.0 pg/ml (norm: < 7.0 pg/ml). Laboratory findings are summarized in Table 1. Antibiotic treatment with ampicillin and gentamicin was started. Echocardiography performed on the second day of life showed normal heart anatomy and no pericardial effusion. With increasing distension of the bowel with no signs for perforation or intramural gas in the x-ray (Fig. 1), a laparotomy was performed on the second day of life, which revealed a partial obstruction of an ileum segment due to adhesions and scar tissue. There was no finding of perforation or malrotation. A partial ileal resection and an ileostomy were performed. Pathologic examination of the resected ileum segment showed inner layer necrosis of the mucosa, mesenteric edema with blood congestion and focal neutrophilic-granulocytic infiltration with focal endothelial proliferation. The establishment of post-operative enteral nutrition was markedly slow, with repeated episodes of very liquid stools and vomiting necessitating a reduction in the amount of enteral nutrition and an increase in the requirement for partial parenteral nutrition. The patient received donor milk on day of life (DOL) one and then breast milk. Due to the recurrent vomiting and poor weight gain, the patient was given a trial of dairy-free formula from DOL 20 to 23. As there was no improvement in symptoms, the diet was switched back to breast milk.

**Table 1:** Laboratory results

Laboratory parameters	Result	Norm	Day of life
C-reactive protein [mg/dL]	8	< 0.5	1
White blood cell count [/µl]	24,400	6000–13300	1
Interleukin 6 [pg/ml]	136	< 7	1
Conjugated bilirubin [mg/dL]	3.44	< 1.0	1
Conjugated bilirubin (maximum) [mg/dL]	28.52	< 1.0	75
Aspartate aminotransferase (maximum) [U/L]	402	< 79	50
Alanine transaminase (maximum) [U/L]	115	< 48	50
Gamma-glutamyltransferase (maximum) U/L	515	< 200	46
Cholinesterase (minimum) [U/L]	1811	3648–10,336	75
Prothrombin ratio (minimum) [%]	46	64–108	75
Ammonia (maximum) [µmol/L]	127	< 48	67
Bile acids [µmol/L]	96	< 25	27
Urinary mevalonic acid [mmol/mol creatinine]	377.4	0–0.84	25
Mevalonolactone [mmol/mol creatinine]	674	0–6	25

Fecal calprotectin was 72 µg/g on DOL 37 (normal range not available for this age).

On DOL eight, the premature infant developed a maculopapular rash, which particularly affected thorax, face, and extremities and lasted for about a week.

**Fig. 2:**
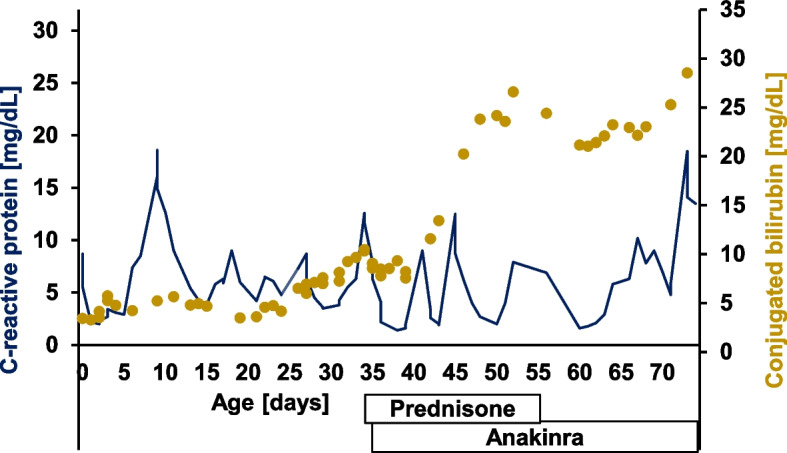
Dynamics of C-reactive protein (CRP) and conjugated bilirubin levels over time. The blue line shows the CRP values, and the yellow dots show the conjugated bilirubin levels over time. Prednisone was started on day of life (DOL) 35 and anakinra was started on DOL 36

She developed cholestasis with elevated conjugated bilirubin of 3.44 mg/dL (norm: < 1.0 mg/dL) directly after birth which increased over time to a maximum of 28.52 mg/dL (Fig. 2). Additionally, she presented with intermittent pale stools. As part of an increasing liver dysfunction a reduced prothrombin ratio (minimum 46%; norm: 64–108%), reduced cholinesterase (minimum 1811 U/L; norm: 3648–10,336 U/L) and slightly increased ammonia (maximum 127 µmol/L; norm: < 48 µmol/L) became apparent. Maximum aspartate aminotransferase was 402 U/L (norm: < 79 U/L) on DOL 50, maximum alanine transaminase was 115 U/L (norm: < 48 U/L) on DOL 50 and maximum gamma-glutamyltransferase was 515 U/L (norm: < 200 U/L) on DOL 46. Bile acids on DOL 27 were elevated with 96.0 µmol/L (norm: < 25 µmol/L). Due to cholestasis ursodeoxycholic acid was started on DOL 27. Sonography revealed hepatosplenomegaly, a small gallbladder with an irregular wall structure and hypoplastic bile ducts. Due to massive ascites, temporary drainage by means of a pigtail catheter was necessary from DOL 40 to 45. Due to anemia, coagulopathy and thrombocytopenia, a total of ten red blood cell transfusions, seven fresh frozen plasma transfusions and one platelet transfusion were necessary. Because of erythema and induration in the area of the peripherally inserted central catheter on the left arm, an ultrasound examination of the vessels was carried out on DOL 33. This revealed a thrombus in the vena subclavia sinistra and in the vena cava inferior. The preterm infant received an antithrombotic therapy with enoxaparin. Antithrombin III was regularly substituted due to low antithrombin III levels. Repeated cranial sonographies showed no abnormal findings. Ophthalmologic examination revealed no retinopathy of prematurity and no evidence of retinitis, uveitis or cataract.

Due to the recurrent CRP elevations (Fig. 2) and clinical correlates of sepsis, multiple antibiotic therapies with changing combinations, including cefotaxime, vancomycin and imipenem were administered. In addition, the central catheters were replaced several times. However, blood cultures always remained sterile. Recurrent thrombophlebitis was seen in the area of the catheters within a few days of new placement.

Based on the chronic inflammation with recurrent CRP peaks (Fig. 2) and progressive clinical deterioration in the context of liver dysfunction and increasing abdominal problems with insufficient enteral nutrition, the urinary mevalonic acid was determined on DOL 25. This was significantly elevated at 377.4 mmol/mol creatinine (norm: 0–0.84 mmol/mol creatinine), which confirmed the diagnosis of severe MKD. Correspondingly mevalonolactone was highly elevated with 674 mmol/mol creatinine (norm: 0–6 mmol/mol creatinine).

Immunoglobulin D levels measured on DOL 23 and 36 were not elevated. An anti-inflammatory therapy with prednisone (maximum dosage 2 mg/kg/d) and anakinra (2 mg/kg/d) was started (Fig. 2). CRP initially fell to a minimum of 1.4 mg/dL after the start of treatment (Fig. 2). However, CRP rose again within a few days, without any clinical correlate for an infection and did not respond to the increase of dosage of anakinra of up to 10 mg/kg/d. 34 days after the start with anakinra mevalonic acid was still elevated at 288.51 mmol/mol creatinine.

In addition to the progressive liver dysfunction, there was also a massive increase in abdominal circumference due to hepatosplenomegaly, ascites and progressively dilated intestinal loops with lactate acidosis and increasingly poor enteral nutrition. Due to the distended abdomen, the infant developed progressive respiratory insufficiency with need for high flow nasal cannula therapy and oxygen. Unfortunately, the patient died at the age of 77 days due to cardiorespiratory failure.

## Discussion

Here we present the case of a preterm infant with severe MKD. To our knowledge this is the first case of a preterm infant with this disease who developed intrauterine intestinal obstruction requiring immediate surgical intervention after delivery. A range of differential diagnoses were considered prior to surgery, including bowel atresia, spontaneous intestinal perforation followed by adhesions, malrotation, volvulus or ileal duplication cyst [14, 15]. However, these diagnoses were not found intraoperatively.

Even though, there is a report of an infant with MKD where prenatal ultrasounds revealed hyperechogenicity and dilatation of the bowel, no immediate surgical intervention was required directly after delivery [12]. Still, this infant developed a megacolon and required an ileostomy during the first months of life [12]. Nimubona et al. described the case of an infant born at 38 weeks of gestation showing a clinically distended abdomen after feeding during the first days of life which persisted over the first months of life besides good feeding and without showing radiological abnormalities. However, at the age of eight months this patient presented with an obstruction of the jejunum and laparotomy revealed inflammatory adhesions requiring adhesiolysis [16]. Bader-Meunier et al. reported on ten patients with MKD and early onset inflammatory bowel disease that manifested between the age of one day up to six years [17]. Surgery was required in seven patients and laparotomy revealed abdominal adhesions (in six patients), bowel perforation (one patient) and sterile peritonitis (one patient) [17]. Median fecal calprotectin was 889 µg/g in these infants [17]. In our patient fecal calprotectin was 72 µg/g on DOL 37 which was not significantly elevated. However, it was only measured once and the values of fecal calprotectin are known to vary considerably between individual preterm infants, which makes interpretation challenging [18]. Overall, intestinal problems are common in MKD and, as our case shows, can lead to severe problems already in utero. Particularly if other recurrent inflammatory symptoms are present, a diagnosis of MKD should be considered in the case of pre- or perinatal intestinal obstruction.

In addition to the intestinal obstruction, the rapidly progressive cholestatic liver disease was a leading problem in our patient. Severe cholestasis has been described before in infants with MKD [10, 19]. Chiu et al. reported on a patient with MKD where liver biopsy showed bile duct paucity [20]. A liver biopsy was not performed on our patient. However, ultrasound imaging revealed hepatosplenomegaly, a small gallbladder with an irregular wall structure and hypoplastic bile ducts. Furthermore, massive ascites leading to respiratory distress has also been described before in one patient reported by Erdol et al. [21]. Due to the patient’s low weight and systemic disease, a liver transplant to treat the liver dysfunction was unfortunately unfeasible.

Patients with a perinatal onset of MKD often have a very severe clinical course and many of them die within the first few months of life [10]. Guidelines for the management of patients with MKD, such as the SHARE recommendations by Lengvári et al. and the German Consensus protocol for the treatment of autoinflammatory syndromes by Hansmann et al. generally do not include specific recommendations for neonates and especially not for preterm infants [4, 22]. This means that experience with the use of anti-inflammatory drugs in neonates with severe MKD is largely based on individual case reports. Corticosteroids have been shown to reduce symptoms and are recommended as part of the on-demand therapy in patients with MKD [4, 22]. As a well-known and established therapy in the autoinflammatory disease of mild MKD, the blockage of Interleukin-1 with anakinra or canakinumab is also a possible treatment option in severe MKD [4, 23]. Even though data is limited anakinra has been shown to be effective in some neonates suffering from MKD [7, 24]. In addition, an on-demand use of anakinra has been shown to significantly shorten relapses [25]. Our patient was treated with anakinra and prednisone but unfortunately did not show a good clinical response to treatment. Canakinumab has been studied in a larger randomized controlled trial to evaluate its efficiency for patients with MKD [26]. Of the patients who received canakinumab, 35% showed a complete response for the primary outcome (resolution of fever flares and no flares until week 16) after 16 weeks [26]. However, as the study only included patients ≥ 2 years of age it is likely that the most severe forms of MKD were not included. There are only individual case reports on the use of canakinumab in neonates, some of which have shown a response to treatment, while others have not [21, 27]. Furthermore, haematopoietic stem cell transplantation has been described as a possible effective treatment in children with MKD in recent years [28, 29]. However, it must be taken into account that the patients reported by Jeyaratnam et al. received their stem cell transplantation at a median age of 2.6 years [29]. Our patient was born prematurely at 30 + 2 weeks of gestation with only 1240 g and had to struggle with severe complications immediately after birth. Due to the rapidly progressive clinical deterioration stem cell transplantation was unfortunately not a viable option for her.

## Conclusion

This report highlights that severe forms of MKD can manifest prenatally, and the diagnosis can be confirmed by amniocentesis with genetic testing. MKD should also be considered as a differential diagnosis in infants with pre-/or postnatal onset of intestinal obstruction especially if associated with recurrent inflammatory episodes or cholestasis. Further diagnostics should be taken in a timely manner to confirm the diagnosis, and an anti-inflammatory therapy should be started at an early stage. However, the management of severe MKD remains challenging, and there is an urgent need for data on adequate treatment for neonates with severe MKD.

## Data Availability

No datasets were generated or analysed during the current study.

## Abbreviations

CRP: C-reactive protein
DOL: Day of life
HIDS: Hyperimmunoglobulinemia D syndrome
MKD: Mevalonate kinase deficiency
MVK: Mevalonate kinase
